# Designing of two dimensional lanthanum cobalt hydroxide engineered high performance supercapacitor for longer stability under redox active electrolyte

**DOI:** 10.1038/s41598-022-06839-8

**Published:** 2022-02-23

**Authors:** Deepa B. Bailmare, Prashant Tripathi, Abhay D. Deshmukh, Bipin Kumar Gupta

**Affiliations:** 1 Energy Materials and Devices Laboratory, Department of Physics, RTM Nagpur University, Nagpur, 440033 India; 2 Photonic Materials Metrology Subdivision, Advanced Materials and Device Metrology Division, CSIR-National Physical Laboratory, Dr. K.S. Krishnan Road, New Delhi, 110012 India

**Keywords:** Supercapacitors, Materials science, Electronic devices

## Abstract

Redox active electrolyte supercapacitors differ significantly from the conventional electrolytes based storage devices but face a long term stability issue which requires a different approach while designing the systems. Here, we show the change in layered double hydroxides (LDHs) systems with rare earth elements (lanthanum) can drastically influence the stability of two dimensional LDH systems in redox electrolyte. We find that the choice of rare earth element (lanthanum) having magnetic properties and higher thermal and chemical stability has a profound effect on the stability of La–Co LDHs electrode in redox electrolyte. The fabricated hybrid device with rare earth based positive electrode and carbon as negative electrode having redox electrolyte leads to long stable high volumetric/gravimetric capacity at high discharge rate, demonstrates the importance of considering the rare earth elements while designing the LDH systems for redox active supercapacitor development.

## Introduction

In pursuit of on demand highly efficient energy storage devices with long cycle stability increasingly employing various new nanostructures and electrolytes systems. The device that fits these demands is hybrid energy storage systems also called supercapacitor or ultracapacitor. The electrochemical energy storage (EES) is playing an important role in defining new age electronic devices. Here, supercapacitors and batteries are the most successful systems on the Ragone chessboard and widely investigated in various field of study^1^. However, the energy that can be stored in the current systems is much lower than batteries, making them unsuitable for many applications^2^. Recently, transition metal layered double hydroxides have been extensively studied due to their variable layered nanostructures, low cost and high specific capacitance with high energy and power density. These materials show interesting performance with different application like oxygen evolution reaction, composites and supercapacitors acts as an electrolyte reservoir, but lacking in thermal and chemical stability in various electrolytes^3^. There have been numerous attempts takes place to improve the qualitative performance of layered double hydroxide^4–9^ but still limits its practical application due to instability in redox electrolytes. Therefore, there is an urgent need for a new electrode system which can meet the demand. Replacing the M^2+/^M^3+^ (metal cation) in LDHs with rare earth element lanthanum (La) could result devices with higher stability. Lanthanum is known to be the most efficient rare earth element having excellent electric, magnetic properties and lightest in weight in rare-earth element in periodic table, hence making it suitable for storage applications. Moreover, knowing the fact that the rare-earth materials in its trivalent states exhibits great stability in wide range of temperature, hence ensure the thermal and chemical stability^10–12^. Materials with intrinsic magnetic behaviour resembles the effect of magnetism when placed in a magnetic field, the electrode material results in enhancement of current due to the convection flow near the electrode surface and reducing the contact resistance between electrode and electrolyte interface and hence increasing the stability. The previous reports examined the magnetic materials for La^+^/Na^+^ batteries^9^ to some extent, however, these systems not been considered for the supercapacitor applications.

In this study, we demonstrate the choice of rare earth material and their intrinsic magnetic behaviour plays a significant role in stability of the supercapacitor device in redox electrolyte. The comparative charge storage mechanism in alkaline and redox active electrolyte has been proposed. The LDH shows the dominance of non-diffusion controlled behaviour in alkaline electrolyte. These results are contrary to expectations based on LDHs, an electrode for supercapacitor, where redox electrolyte with highest capacitance is achieved but the stability of the device remains a challenge. Therefore, the rare earth element with magnetic behaviour should be a best choice for a redox active electrolyte supercapacitor device. We show that the rare earth element (lanthanum) enabled LDHs electrode may be a proper choice for achieving superior performance of supercapacitor in redox electrolyte.

## Results

### Design of electrode and structural characterization

**Figure 1:**
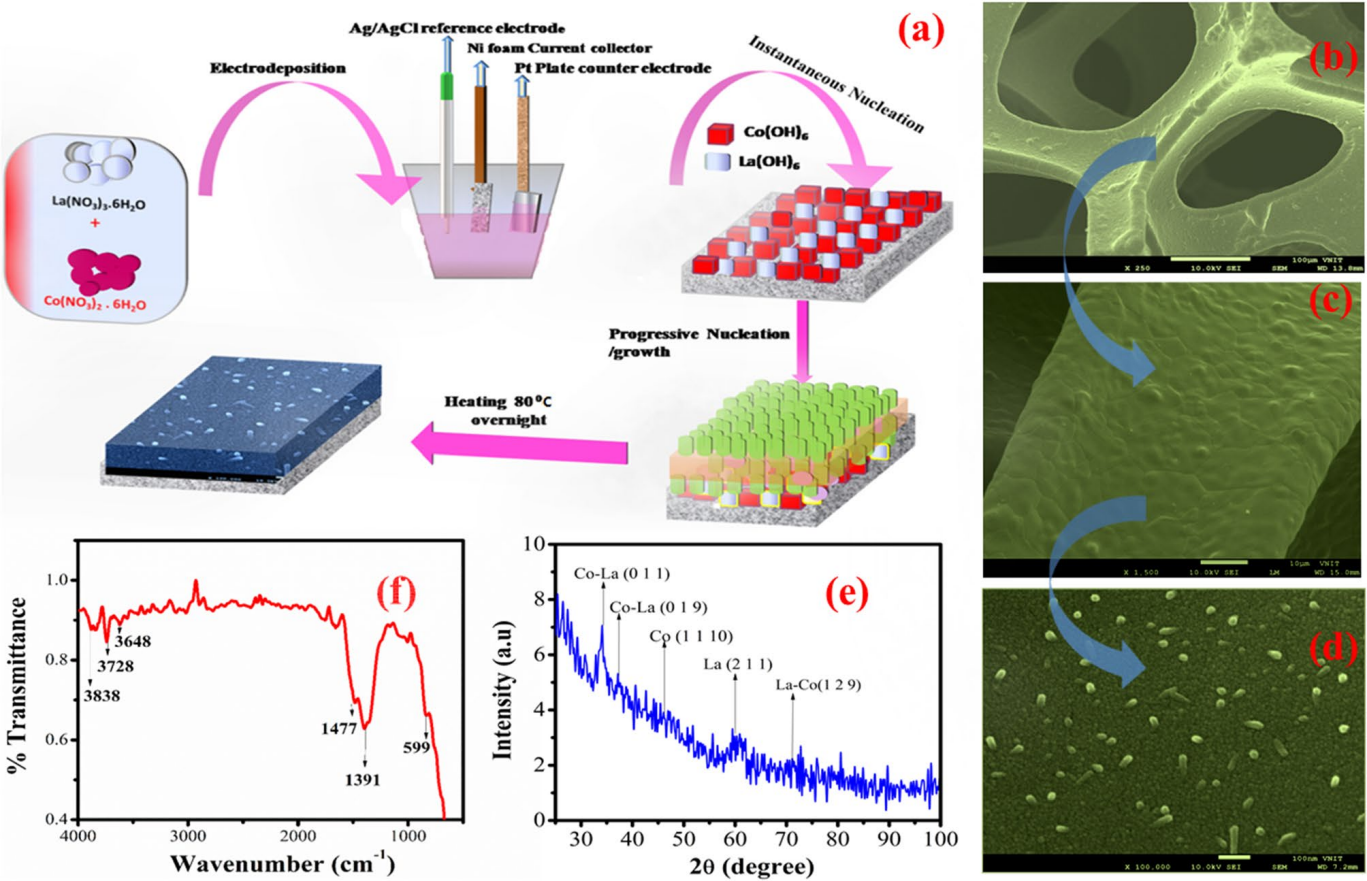
(**a**) Schematic diagram of formation of La–Co layered double hydroxides on Ni foam substrate, (**b–d**) shows the FESEM images of prepared La–Co LDH grown on Ni foam substrate, (**e**) X-ray diffraction spectra of La–Co LDH material, (**f**) FTIR spectroscopy of La–Co LDH material sheds the light on surface bonding and functionality present in the material.

To facilitate redox electrolyte for LDHs based supercapacitors, a rare earth based layered double hydroxide system (La–Co LDHs) was used as the working electrode. Figure 1a shows the schematic representation of the developed two-dimensional La–Co layered double hydroxide nano architecture. The beautiful nanostructure forest spread on the three-dimensional nickel foam was observed by FESEM shown in Fig. 1b–d. The structure of nanorods observed in the present study confirms the chemical growth of nanometer sized hydroxides involved in the process of nucleation. The process followed by partial nucleation finally initiates the growth of nanostructure over a substrate. The presence of lanthanum and cobalt in the double hydroxide were confirmed by the EDAX facility with FESEM (Fig. S1). The X-ray diffraction (XRD) patterns were also recorded for La–Co layered double hydroxides as shown in Fig. 1e. The XRD patterns are well in agreement with the simulated XRD pattern of individual single crystal data of lanthanum hydroxide and cobalt hydroxides confirmed with the JCPDs crystallographic database. The well-defined matching ( 0 1 1), (0 1 9), (1 1 10) and (1 2 9) peaks indicate the successful formation of layered lanthanum cobalt double hydroxides. Further, the double hydroxide analyzed with the help of Fourier Transform Infrared Spectroscopy (Fig. 1f) illustrates the well-defined peak in the range of 1000 cm^−1^ to 1500 cm^−1^ due to the lattice vibration of metal–oxygen bond. The peak at higher frequency side gives the metal–oxygen bonding through the stretching mode, mainly the peaks located at higher frequency region of 3500 cm^−1^ to 4000 cm^−1^ confirms the stretching vibrations of physically absorbing peaks of OH bonding with metal ions on to a substrate, hence showing the metal hydrogen bonding of lanthanum cobalt double hydroxides material.

### Electrochemical characterization

**Figure 2:**
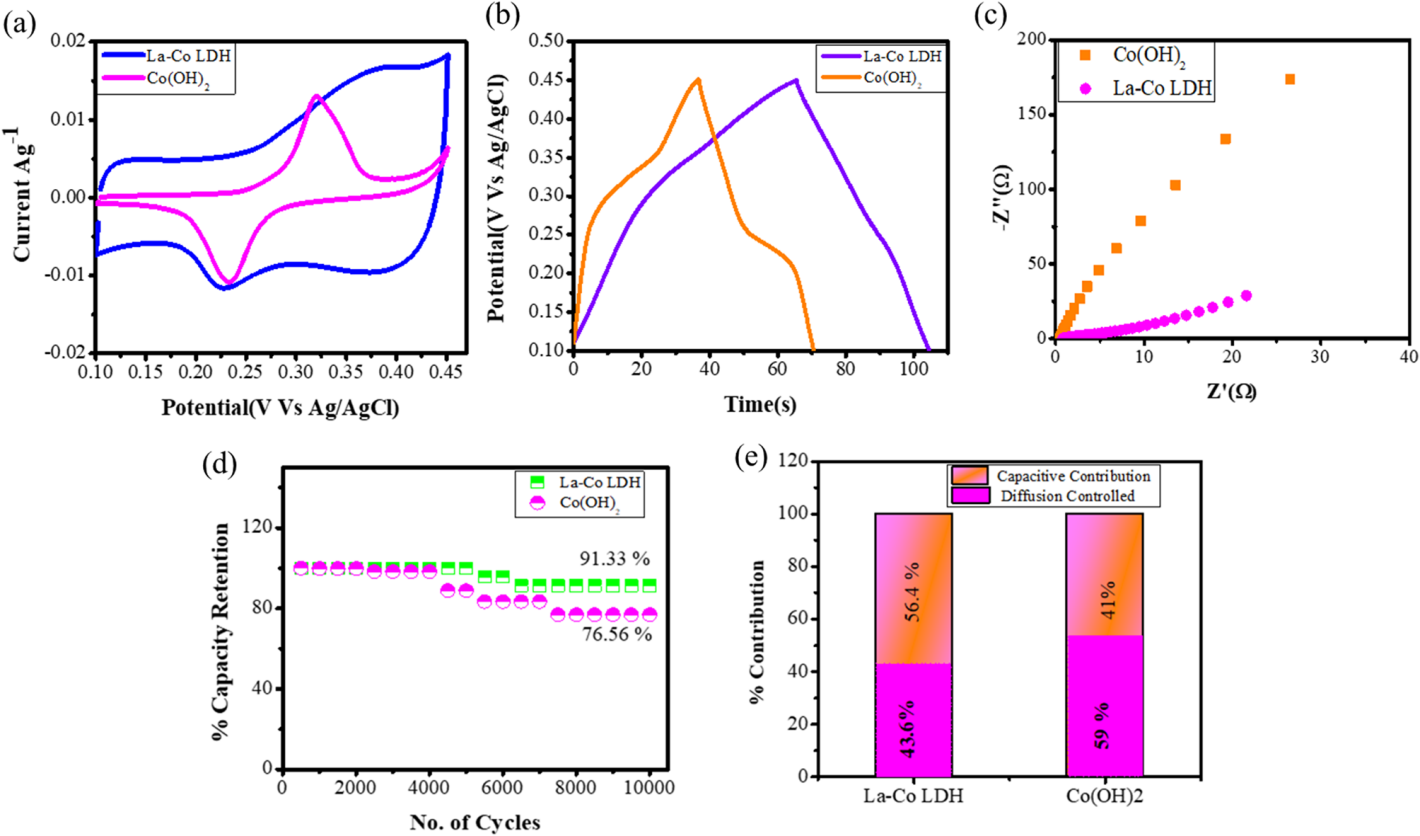
(**a**) Comparative CV curve of La–Co LDH with Co(OH)_2_ at 10 mVs^−1^ scan rate, (**b**) Comparative charge discharge characteristics of La–Co LDH with Co(OH)_2_ at 5 A g^−1^ current density, (**c**) Comparative Nyquist plot of La–Co LDH and Co(OH)_2_, (**d**) cyclic stability characteristics of La–Co LDH and Co(OH)_2_ in alkaline electrolyte (2 M KOH electrolyte), (**e**) differentiation of capacitive and diffusion controlled contribution from CV curve at 10 mVs^−1^ scan rate.

To facilitate the more fundamental understanding of the rare earth based LDHs, electrochemical characteristics of La–Co LDHs system were compared with Co(OH)_2_through cyclic voltammetry (CV), Galvanostatic charge Discharge measurement (GCD) and electrochemical impedance spectroscopy (EIS). The effect of electrodeposited La–Co nanostructured double hydroxide in 2 M KOH alkaline electrolyte was firstly investigated. Mostly the metal oxides/hydroxides carry out transfer of charge at electrode and electrolyte interface with fast redox reaction and absorption/desorption of ions^13^. The well-defined redox peaks are obtained at anodic and cathodic peaks within a potential window of 0.1 V to 0.45 V observed in Fig. S2a. The pair of well-defined redox peaks indicates the significant faradic behaviour of prepared electrode material. Meanwhile, cobalt hydroxide in alkaline electrolyte also exhibits pseudocapacitance rooted in the redox reaction. During the reaction process at charging cobalt hydroxide gets oxidized to CoOOH, whereas for the discharge the reaction is reversed. A comparative CV curve of La–Co LDH and Co(OH)_2_ is shown in Fig. 2a. This implies strong pseudocapacitive behaviour of Co(OH)_2_ with a pair of redox peaks at upper and lower peak positions unlike La–Co LDH. The figure indicates doping of lanthanum give the peak shift towards ~ 0.32 to ~ 0.40 indicates the existence of variable oxidation state of lanthanum and cobalt in layered double hydroxides. Mostly, rare earth metal ions get attached to the crystal lattice of metal hydroxides for improving ionic conductivity and structural stability. The large ionic radii of La^3+^ metal ion creates a charge imbalance with uncertainty in the hydroxide lattice to improve the chemical activity of overall electrode material^14^. Despite their propensity to form a stable oxidation state of cations together with the large ionic radii, a lanthanum does not closely resemble cobalt ions. The different reaction mechanism of both the electrodes through CV curve is given below as^15,16^:

1 $${\text{CoOOH }} + {\text{ OH}}^{ - } \leftrightarrow {\text{ CoO}}_{{2}} + {\text{H}}_{{2}} {\text{O }} + {\text{ e}}^{ - } ,$$

2 $${\text{LaOOH}} + {\text{ OH}}^{ - } \leftrightarrow {\text{ La}}\left( {{\text{OH}}} \right)_{{2}} + {\text{ e}}^{ - } .$$

Larger integral area under the CV curve of La–Co LDH indicates larger specific capacity. Moreover the CV curve of both the electrodes possess identical characteristics only the peak shift of La–Co LDH attributed due to the polarization of electrode material in electrolyte interface^16^. Here, all the calculations were carried out in terms of capacity ${(\text{Cg}}^{-1})$ instead of capacitance ${(Fg}^{-1})$. The charge delivered at 5 A g^−1^, calculated using discharge curve in Fig. S2b, is 195 ${\text{Cg}}^{-1}$ in the potential range 0.1 V to 0.45 V in alkaline electrolyte. Figure 2b shows visible difference in La–Co LDH and Co (OH)_2_ charge discharge profile at 5 A g^−1^ current density. The Co(OH)_2_ electrode material possesses 170 C g^−1^ at 5 A g^−1^ which is in good agreement with CV characteristics of Co(OH)_2_ and La–Co LDH. The discharge capacity for different current densities from 5 to 35 A g^−1^ (Fig. S2b) is summarized in Supplementary Table T1. Even at high current density of 35 A g^−1^, it shows the excellent rate capability of (93.36%) than Co(OH)_2_ (82.45%) to its initial capacity shown in Fig. S2d.which correlated the structural stability of electrode and better ion transport ability even at higher current densities. The higher current density profoundly affects the stability of the electrode, but here, the La–Co LDH shows excellent stability of 10,000 cycles with 91.33% capacity retention at 25 A g^−1^, Whereas Co(OH)_2_ in alkaline electrolyte shows stability of 10,000 cycles with 76.56% capacity retention Fig. 2d. The addition of rare earth elements into hydroxides effectively increases cyclic stability with lower energy levels. This can help hydroxides to intercalation/deintercalation of ions easily. The rare earth based elements significantly improves electrical conductivity and efficiency of proton diffusion^17^. Also, its 4f electronic shell structure is very efficient it contains lower energies and increased effective charge draws electrons closer and goes from one element to another. Further the electronic properties of lanthanum resemble with cations and anions of electrolytes and hence, enhances the stability of La–Co LDH even at high discharge current density.

Likewise, the resistive performance of Co(OH)_2_ exceeds La–Co LDH shows excellent ionic polarization of prepared electrode material over cobalt hydroxide Fig. 2c. The capacitive contribution of electrode material possesses fast charge transfer kinetics at electrode and electrolyte interface and provides high stability even at large current density. Hence a large proportion of capacitive response enables high stability and rate capability. Meanwhile La–Co LDH and Co(OH)_2_ give capacitive response of 56.4% and 41% with diffusion controlled response of 43.6% and 59% respectively, Fig. 2e. The quantitative diffusion characteristics of La–Co LDH and Co(OH)_2_ is analyzed by Dunn’s method. This method enables formation of surface capacitive contribution and diffusion controlled contribution at different scan rates with applied potential. At fixed potential a varying current can be expressed with the equation^18^:

3 $${\text{I}}\left( {\text{V}} \right) \, = {\text{ K}}_{{1}} \left( {\text{v}} \right) \, + {\text{ K}}_{{2}} \left( {\text{v}} \right)^{{{1}/{2}}} ,$$

where, K_1_ is capacitive factor and K_2_ is diffusion controlled factor and v is scanning rate mVs^−1^. Plotting i(V) v^1/2^ vs v^1/2^ with linear relationships enables the contribution of capacitive and ion diffusion with slope and intercept of the linear relationship respectively. The capacity of surface contribution and diffusion controlled contribution is examined through the ratio of area shaded to the blank region inside the CV curve (Fig. S3). For this analysis a low scan rate with more points was selected due to the high capacitive response at low current density^17^. The result suggests that although the charge storage responses of La–Co LDH and Co(OH)_2_ are very close, the presence of lanthanum increases the capacity, rate capability and cyclic stability. Since, ion transport at electrode and electrolyte was not hindered at high current rate and still remained the same; such case is consistent with the cyclic stability and rate capability of La–Co LDH at high current density of 25 A g^−1^.

**Figure 3:**
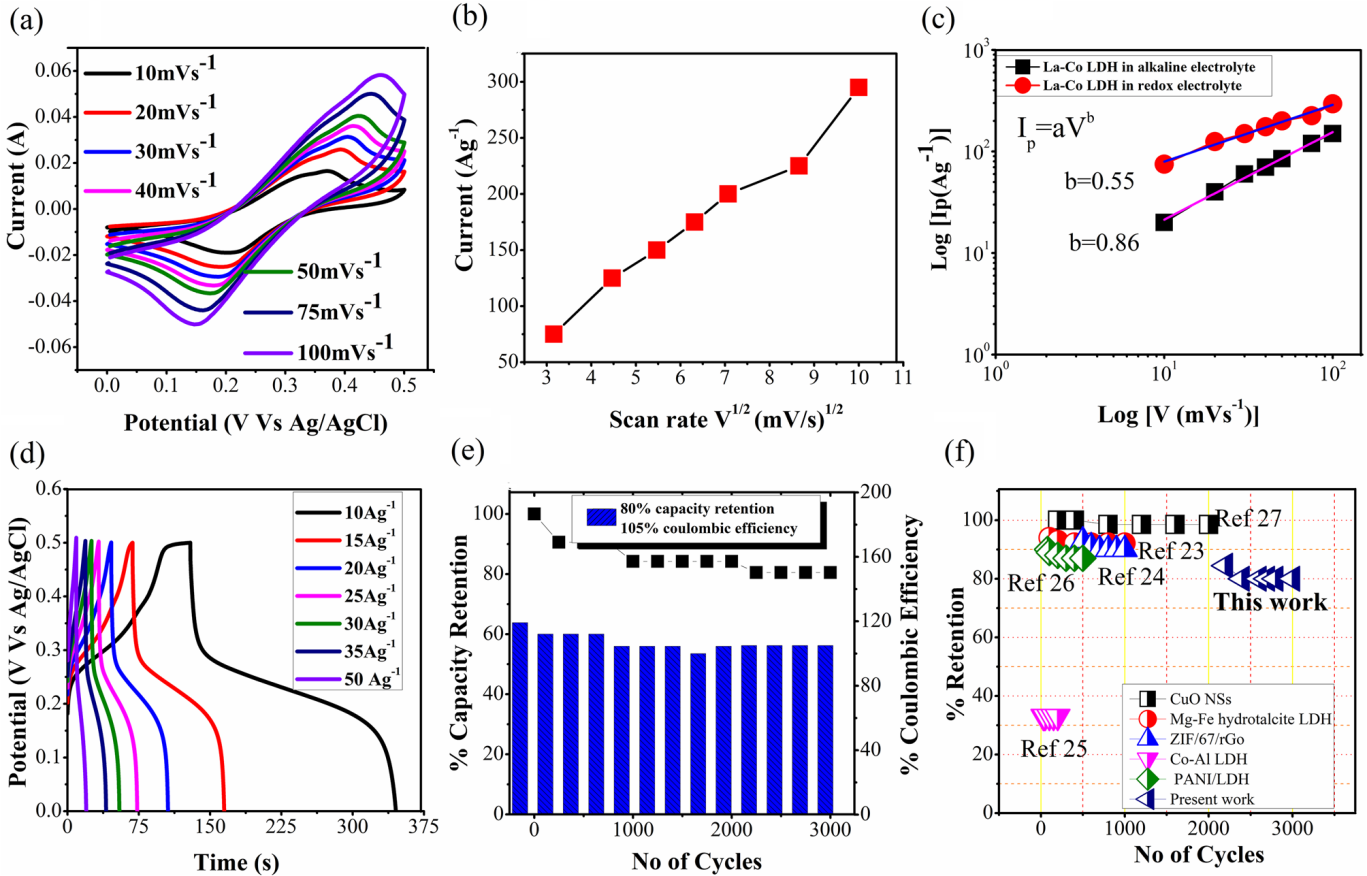
(**a**) CV cyclic voltammetry of La–Co LDH with redox electrolyte, (**b**) peak current vs scan rate V^1/2^ plot of prepared electrode material in redox electrolyte, (**c**) separation of capacitive and diffusion controlled contribution with Power law, (**d**) Galvanostatic charge discharge measurement at various current densities in redox electrolyte, (**e**) cyclic stability of La–Co LDH electrode material in redox electrolyte, (**f**) the comparative cyclic stability plot of La–Co LDH with reported LDH based materials tested in redox electrolyte^23–27^.

In general, choice of electrolyte is important for the charge storage capability as well as defines the potential window. To further improving charge storage capability of our electrode, redox active species in alkaline electrolyte were added and electrochemical performances were evaluated. The additive concentration is an important factor which decides the performance and stability of electrode. Considering this parameter, 0.05 M of redox active species were added to alkaline electrolyte and cyclic voltammetry of La–Co LDH and Co(OH)_2_ were shown in Fig. 3a and Fig S4a. Two independent redox peaks were observed in CV curve resembles two redox reaction takes place simultaneously in between ${M}^{2+}/{M}^{3+}$ and ${Fe(CN)}_{6}^{3-}/{Fe(CN)}_{6}^{4-}$ in electrode and electrolyte interfaceSince the effect of lanthanum and cobalt stimulates due to the synergism. Also, to analyze the effect of La in La–Co LDH, a comparative cyclic voltammetry analysis of La–Co LDH with Co(OH)_2_ in redox electrolyte was performed. Figure S4b clearly shows the better current carrying capability with La doping. The reversible redox reaction is described as follow, where M represents La/Co material:

4 $${M}^{2+/3+}+{e}^{-}\leftrightarrow {M}^{3+/4+},$$

5 $${Fe(CN)}_{6}^{4-}{- {e}^{-} \leftrightarrow Fe(CN)}_{6}^{3-},$$

The linear relation between $i(A)$ and square root of scan rate ${V}^{1/2}{(\text{mV}/\text{s})}^{1/2}$ for redox electrolyte shows exactly the reverse relationship compared to alkaline electrolyte, demonstrating the dominance of diffusion controlled process Fig. 3b. Comparative charge storage mechanism for alkaline electrolyte and redox active electrolyte were examined for various scan rates shown in Fig. 3c which reveals the linear relationship for both the electrolytes. Power law $I={aV}^{b}$ is used to better understand the role of the electrolyte solvents influencing the electrochemical performance; where $a\, \text{and}\, b$ are constants. Value of b confirms the charge storage mechanism, where b = 0.5 displays diffusion controlled processes whereas b = 1 displays capacitive contribution of the electrode^19,20^. Interestingly, the difference in electrochemical signature and kinetics is observed; for alkaline electrolyte the b values calculated to be 0.86 suggesting the small diffusion controlled contribution whereas for redox active electrolyte it is 0.55 confirming the diffusion controlled process. These results are much different from the ideal layered double hydroxides behavior in alkaline electrolyte. To further validate the results, It is also determined by the formula^14^; ${f}_{b}=(1-b)/0.5,$ illustrating the dominance of diffusion process in redox electrolyte with value of ${f}_{b}=0.9.$ As the behaviour of electrode shows diffusion controlled faradic response which is similar to battery type material, therefore the specific capacity estimated by equation^21,22^ (given in Supplementary Information).

Figure 3d and Fig. S4c shows the GCD plot of La–Co LDH and Co(OH)_2_ with redox electrolyte. The discharge capacity for La–Co LDH and Co(OH)_2_ was calculated to be 2162 C g^−1^ and 1108.4 C g^−1^ at 10 A g^−1^ current density which is much higher than alkaline electrolyte, the comparative performance of La–Co LDHs and Co(OH)_2_ in both the electrolyte shown in Figs. S4d and S5 and summarized in Supplementary Table S1. The distinct electrolyte environment between electrode and electrolyte interface not only influences the charge storage mechanism but also drastically influences the amount of charge to be stored. Previously reported strategies for increasing the charge storage capability of LDH materials have focused on redox electrolytes, however, the rate capability is much lower than alkaline electrolyte which may attribute to very less intercalation/de-intercalation at surface and cannot utilize the bulk material which also results in lower cycling stability. But, the choice of rare earth element in this study offers higher charge transport and chemical stability compared to other LDHs systems. This insertion of a rare earth element with cobalt metal ion uplifted the rate and cyclic properties of electrode material. A rare earth-based material mainly gets affected via inter structural distortion of existing compounds. A partial incorporation of 2^+^ unlike (Co^2+^, Ca^2+^, Sr^2+^etc.) oxidation state containing diverse cations for La^3+^ species leads to give large number of oxygen vacancies in the structure and give diffusivity along with the good electrochemical behavior^28^. On comparison, without La doping Co(OH)_2_ shows less capacity retention of 64.67% over 3000 cycles as shown in Fig. S4e. The highest cycling stability of La–Co LDH is achieved in the redox electrolyte with 80% retention after 3000 cycles, which contrast previous reports, summarized in Supplementary Table T2. However, the coulombic efficiency of redox electrolyte is also higher 105% compare to alkaline electrolyte 92% (Fig. S6, Fig. 3e), because discharge time in case of redox electrolyte is higher than that of charging time in consistent with earlier reports^28^. The comparative stability report of previous report with this study distinguished the choice of LDHs system is important, shown in Fig. 3f. Therefore, choice of LDH system is a critical for improving the performance in redox electrolyte. To further understand the effect of redox additives in alkaline electrolyte, electrochemical impedance spectroscopy (EIS) measurements were performed for both the electrolyte at 0.035 V open circuit voltage. Nyquist plot presented in Fig. S7 shows the response over 10^4^ Hz to 10^−1^ Hz. Addition of redox active species decreases the charge transfer resistance but slightly increase in series resistance observed as compared to the alkaline electrolyte Fig. S2c. The quasi-vertical curve in the low frequency region Fig. S7 signifies the capacitive behaviour, as the ionic and electronic charge accumulate on the surface of micro pores with formation of electric double layer ensure the elegant transport of ions across the micro pores. Further, a less charge transfer resistance suggests fast electron transfer during the redox reaction. This evidence that the redox active species enhances the accessibility of surfaces within the nano-porous LDH structure is inconsistent with what we have expected^29^.

### Design of hybrid supercapacitor device

**Figure 4:**
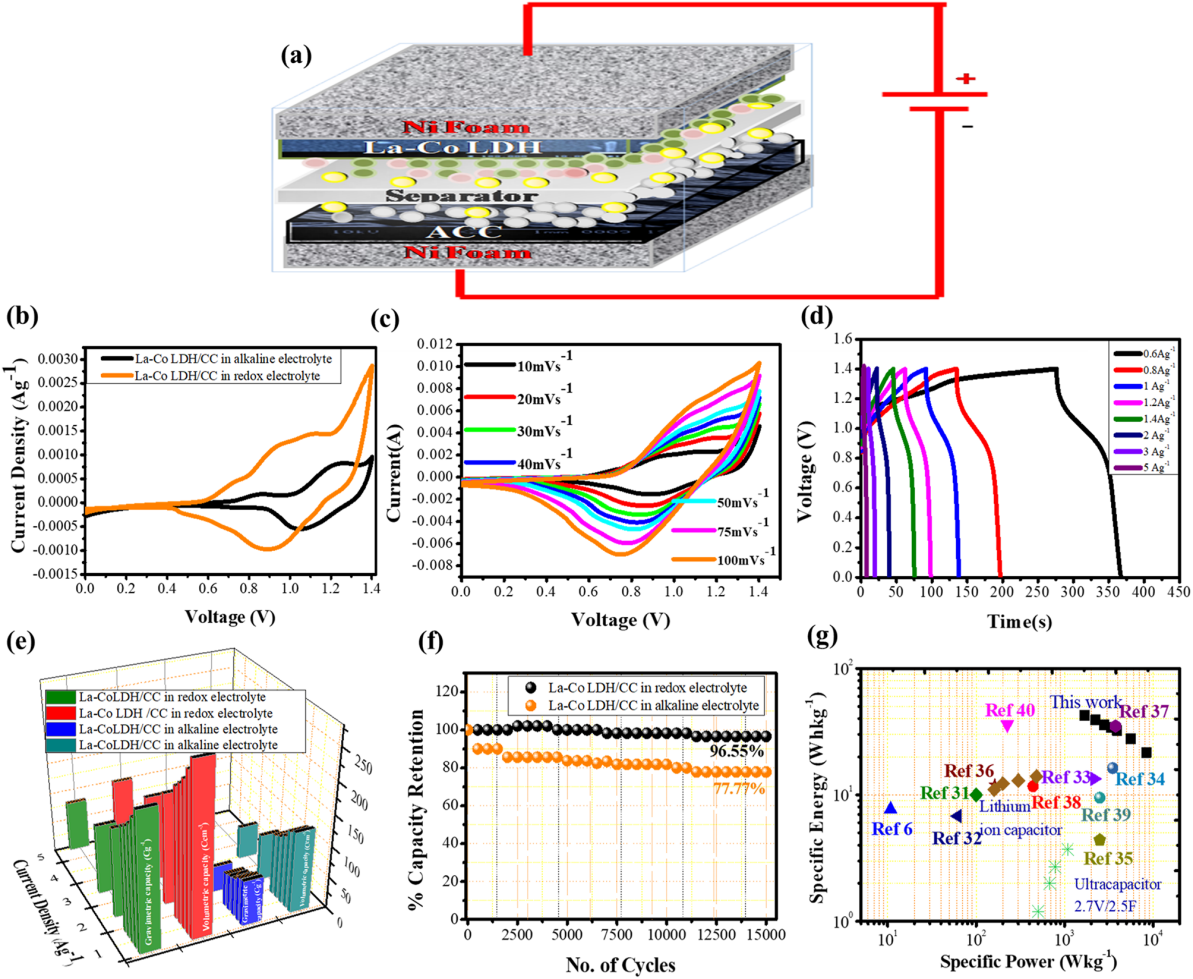
(**a**) Schematic representation of two electrode cell fabricating by using La–Co LDH/CC, (**b**) comparative cyclic voltammetry curve of La–Co LDH/CC in alkaline and redox electrolyte, (**c**) CV curve of La–Co LDH/CC in redox electrolyte, (**d**) Galvanostatic charge discharge process at various current density, (**e**) comparative Gravimetric and Volumetric capacity of La–Co LDH/CC in redox electrolyte and alkaline electrolyte, (**f**) capacity retention plot of La–Co LDH/CC showing high cyclic stability at redox electrolyte compare to alkaline electrolyte, (**g**) comparative Ragone plot of specific energy and specific power of La–Co LDH/CC with reported literatures ^31–40^.

The rare earth based LDHs system was further explored for hybrid device, enlightening significantly enhanced electrochemical properties at small redox additives. A full electrochemical performance of a La–Co LDH as positive electrode and activated carbon cloth (ACC) as negative electrode in supercapacitor device was conducted using redox active electrolyte to demonstrate the practical applicability of this system. The ACC electrode displays excellent electrochemical behavior with 0.0–1.0 V. The symmetric cell of ACC in redox electrolyte displays electrochemical double layer behaviour with quasi rectangular shape. The specific capacity of the ACC electrode could be calculated from the GCD plot, which was found to be 110.32 C g^−1^ at 0.3 A g^−1^ current density. The comprehensive study of ACC two electrode cells, CV analysis, GCD measurement and electrochemical performance is provided in Fig. S8. Hence, tunning excellent properties of ACC and La–Co LDH in redox electrolyte with ACC (0.0–1.0 V) and La–Co LDH (0.0–0.5 V) are beneficial for hybrid supercapacitor. The use of this concept gives the good synergistic interaction of both the electrodes in hybrid device. In Fig. 4b, a comparative CV curve of La–Co LDH/CC in alkaline and redox electrolyte shows commendable performance of hybrid device in redox electrolyte. The device took good area under the curve with high current response in redox electrolyte. In Fig. 4c, the CV curves for a different scan rate from 10 to 100 mVs^−1^ in the voltage  window of 0 V to 1.4 V. The significant nature with slight distortion was observed due to the effect of faradic and non-faradic reaction between electrode and electrolyte interfaces. As the hybrid device displays the electrochemical performance in between supercapacitors and battery type electrodes^30^. The schematic arrangement of a fabricated device shown in Fig. 4a, where mass loading on both electrodes is dependent upon the optimal mass ratio of each electrode according to Eq. (6), which is found to be 1:0.3 for negative and positive electrodes, respectively. The total charge stored (Cg^−1^) in the La–Co LDH/CC in redox electrolyte was calculated using GCD curves at various current densities shown in Fig. 4d. Further, the performance of La–Co LDH/CC in alkaline and redox electrolyte was compared for analyzing the suitability of the prepared device as shown in Fig. 4e. The capacity of the La–Co LDH /CC in alkaline electrolyte and redox electrolyte were 72.67 C g^−1^ and 218.4 C g^−1^ at 0.6 A g^−1^, respectively. The detailed electrochemical study of La–Co-LDH/CC in alkaline electrolyte is shown in Fig. S9. Also, a comparative electrochemical performance of La–Co-LDH in alkaline and redox electrolyte is shown in Supplementary Table T3.

The volumetric capacity at various current densities of La–Co LDH/CC in redox electrolyte was also summarized in Supplementary Table T4 and found to be 271.02 C cm^−3^ at 0.6 A g^−1^ demonstrating the excellent specific and volumetric capacity together Fig. 4e. The stability of the asymmetric device was tested at 4 A g^−1^ with a voltage window of 1.4 V. The device showed an excellent cycling stability of 96.55% capacity retention over 15,000 cycles Fig. 4f, whereas, in alkaline electrolyte it delivers 77.77% capacity retention. This suggests high cyclic stability of prepared La–Co LDH/CC in redox electrolyte. The EIS measurement was also taken in the frequency range of 10^–1^ Hz to 10^4^ Hz with well fitted simplified equivalent circuit shown in (Fig. S10) which demonstrates very less Rs value of 0.94 Ω in higher frequency region and CPE shows electric double layer capacity of the device. The addition of redox additives further decreases the charge transfer resistance with increase in ionic conductivity. It can be attributed that, the introduction of redox species in the electrolyte gives the synergistic effect and affects the performance of the overall device where redox additive with smaller size give large extent of ions with deep absorption at electrodes surfaces and generates extra faradic reaction in electrode and electrolyte interface with increased stability^41^. Ragone plot shown in Fig. 4g in comparison with previous studies on electrodes with redox electrolyte, demonstrating the improvements that the rare earth based LDH system in the redox electrolyte has made over previous reports. A hybrid device with redox active electrolyte yields a very high energy density of 42.46 Wh kg^−1^ (52.69 mWh cm^−3^) at power density of 1687.15 W kg^−1^ (2093.64 mWcm^−3^). The favorable highly stable redox active electrolyte enabled rare earth based LDH demonstrates how the choice of the right system of electrode and electrolyte can lead to practical application of the final device.

## Discussion

The design and synthesis of rare earth based LDHs makes a cost effective and facile approach towards assembling of supercapacitors with high specific capacity and chemical stability. The La–Co LDH not only delivers good electrochemical performances in alkaline electrolyte but the impact of performance in redox active species makes it a promising candidate for high performance electrochemical applications. The La–Co LDH nanosheets deliver high rate and stability performances in alkaline electrolyte as 10,000 cycles with 91.33% capacity retention and 93.36% rate performances in 10 to 35 A g^−1^ current density. Further, The La–Co LDH reaches a high specific capacity of 2162 C g^−1^ at high current density 10 A g^−1^ with very low resistance in redox electrolyte. The LDHs robustness and structural stability makes it suitable for redox electrolyte. Undoubtedly, La–Co LDH produces high cyclic stability of 15,000 cycles with 96.55% capacity retention in a cell with redox electrolyte and hence developed a scalable approach towards the future LDH materials in supercapacitors. Again, to the best of our knowledge the usage of rare earth is unique for the fabrication of LDH based materials in supercapacitors. Remarkably, the redox active electrolyte based La–Co LDH system brings a highly stable environment near the electrode and electrolyte interface resulting in high specific and volumetric capacity for extended cycling test with minimized charge transport resistance. Thus, this work established the new material for the sake of improving performances of LDHs and hence had great potential to be used in high performance supercapacitor applications.

## Methods

### Materials

All starting materials are taken form Merck or Sigma Aldrich and hence used without further purifications. For experimental purpose, cobalt nitrate hexahydrate (Co(NO_3_)_2_·6H_2_O, Lanthanum(III) nitrate hexahydrate (La(NO_3_)_3_·6H_2_O 99.9%, hydrochloric acid reagent grad 37%, potassium hydroxides pallets were purchased from Merck India private limited. Activated carbon cloth was purchased from Environ care product India limited, which was further etched by using nitric acid solution.

#### Preparation of rare earth enabled La–Co LDH electrode

Two dimensional La–Co LDHs produced by facile electrodeposition method; this process can easily create 2D structure with porous film over a substrate. Here we used 3D porous Ni foam substrate as the current collector. Before electrodeposition, Ni foam was cleaned and degreased with 1 M HCl and deionized water through sonication for about 15 min to remove the oxide layer and surface impurity of Ni foam and then dried in the oven at 80 °C overnight. The La–Co precursor solution was prepared by dissolving Lanthanum nitrate (6 mM) and cobalt nitrate hexahydrate (12 mM) in 30 ml double distilled water under continuous stirring for 30 min. The prepared precursor solution was then subjected to an electrodeposition process. The process takes place in three electrode cells with Ni foam as current collector, Ag/AgCl as reference electrode and platinum plate as counter electrode respectively. The deposition time for the material was taken to be 300 s at − 1.0 V of potential. The instantaneous and progressive nucleation was observed with respect to time. The coated sample was then rinsed with double distilled water each time then placed in a vacuum oven overnight at 80 °C. The overnight heating provides excessive time for material to grow interestingly on 3D porous Ni foam substrate. Similar process is followed for the bare cobalt hydroxide preparation.

#### Preparation of redox electrolyte

In this study, the redox electrolyte is prepared by potassium ferrocyanide as a redox additive in 1 M KOH alkaline solution. In a typical preparation process 0.05 M potassium ferricyanide (K_3_(Fe(CN)_6_) is added in 1 M potassium hydroxide (KOH) in 30 ml double distilled water under continuous stirring for 5 min. The homogeneous solution is obtained which is taken as a redox electrolyte for further study.

##### Fabrication of redox active electrolyte supercapacitor device

Supercapacitor device is fabricated with La–Co LDH as positive electrode material and Activated Carbon Cloth (ACC) as negative electrode material with Celgard separator in redox electrolyte. The mass ratio between positive and negative electrode is calculated by the charge balancing equation with Q+  = Q−, in order to satisfy the charge balance equation, the mass of active materials can be taken like this:

6 $$\frac{m+}{m-}= \frac{C+ \Delta V+}{C-\Delta V-},$$

where, C+ and C− is the specific capacity in C g^−1^ of positive and negative electrode materials.

##### Material characterization

The crystalline structure of prepared La–Co LDH electrode materials was analyzed by PAN- analytical X-ray diffractometer with Cu Kα radiation with proportional detector. The structure and morphologies of La–Co LDH were observed by Field emission scanning electron microscope (JEOL JSM 7610F FEGSEM). The chemical composition and element detection on Ni foam substrate carried out by FTIR analysis.

##### Electrochemical measurements

The La–Co LDH electrode material exposed to electrochemical performance measured by CV (Cyclic Voltammetry), GCD (Galvanostatic Charge Discharge) and EIS (Electrochemical Impedance Spectroscopy) using Metrohm AUTOLAB 128N Potentiostat in three electrode system in alkaline and redox active electrolyte. The three-electrode setup consists of a prepared nanostructured La–Co LDH as working electrode immersed in electrolyte, and Ag/AgCl as reference electrode and platinum plate as counter electrode. The EIS measurements were carried out in the frequency range 0.1 Hz to 10 kHz. The detailed calculations for the electrochemical measurement were given in the Supplementary Information. For device measurement, the nanostructured material was tested for two electrode setups as well with activated carbon cloth as negative electrode and La–Co LDH as positive electrode in redox electrolyte, for the purpose of making two electrode cells we first activated nanostructure La–Co LDH with CV cycling with voltage window 0.0–0.5 V.

## Supplementary Information

Supplementary Information.

## Abbreviations

SC: Supercapacitor
LDHs: Layered double hydroxides
ACC: Activated carbon cloth
CV: Cyclic voltammetry
ASC: Asymmetric supercapacitor
EIS: Electrochemical impedance spectroscopy
GCD: Galvanostatic charge discharge
