# Supplementary material for: Designing of two dimensional lanthanum cobalt hydroxide engineered high performance supercapacitor for longer stability under redox active electrolyte
Source: Sci Rep. 2022 Feb 23;12:3084. doi: 10.1038/s41598-022-06839-8 (PMC8866478; doi:10.1038/s41598-022-06839-8)
Supplement: Supplementary file 1 — Supplementary Information. [file 41598_2022_6839_MOESM1_ESM.docx]

**Supplementary Information**

**Designing of two dimensional lanthanum cobalt hydroxide engineered high performance supercapacitor for longer stability under redox active electrolyte**

Deepa B. Bailmare^1^, Prashant Tripathi^2^, Abhay D. Deshmukh^1*^and Bipin Kumar Gupta^2*^

^1^Energy Materials and Devices Laboratory, Department of Physics, RTM Nagpur University, Nagpur-440033, India

^2^Photonic Materials Metrology section, Advanced Materials and Device Metrology Division, CSIR-National Physical Laboratory, Dr. K.S. Krishnan Road, New Delhi- 110012, India

**Table of content**

| Supplementary Figure S1 | 2 |
| --- | --- |
| Supplementary Figure S2 | 3 |
| Supplementary Figure S3 | 4 |
| Supplementary Figure S4 | 5 |
| Supplementary Figure S5 | 6 |
| Supplementary Figure S6 | 7 |
| Supplementary Figure S7 | 8 |
| Supplementary Figure S8 | 9 |
| Supplementary Figure S9 | 10 |
| Supplementary Figure S10 | 11 |
| Supplementary Table T1 | 12 |
| Supplementary Table T2 | 13 |
| Supplementary Table T3 | 14 |
| Supplementary Table T4 | 15 |
| Supplementary Note | 16 |

**Supplementary Fig 1:**


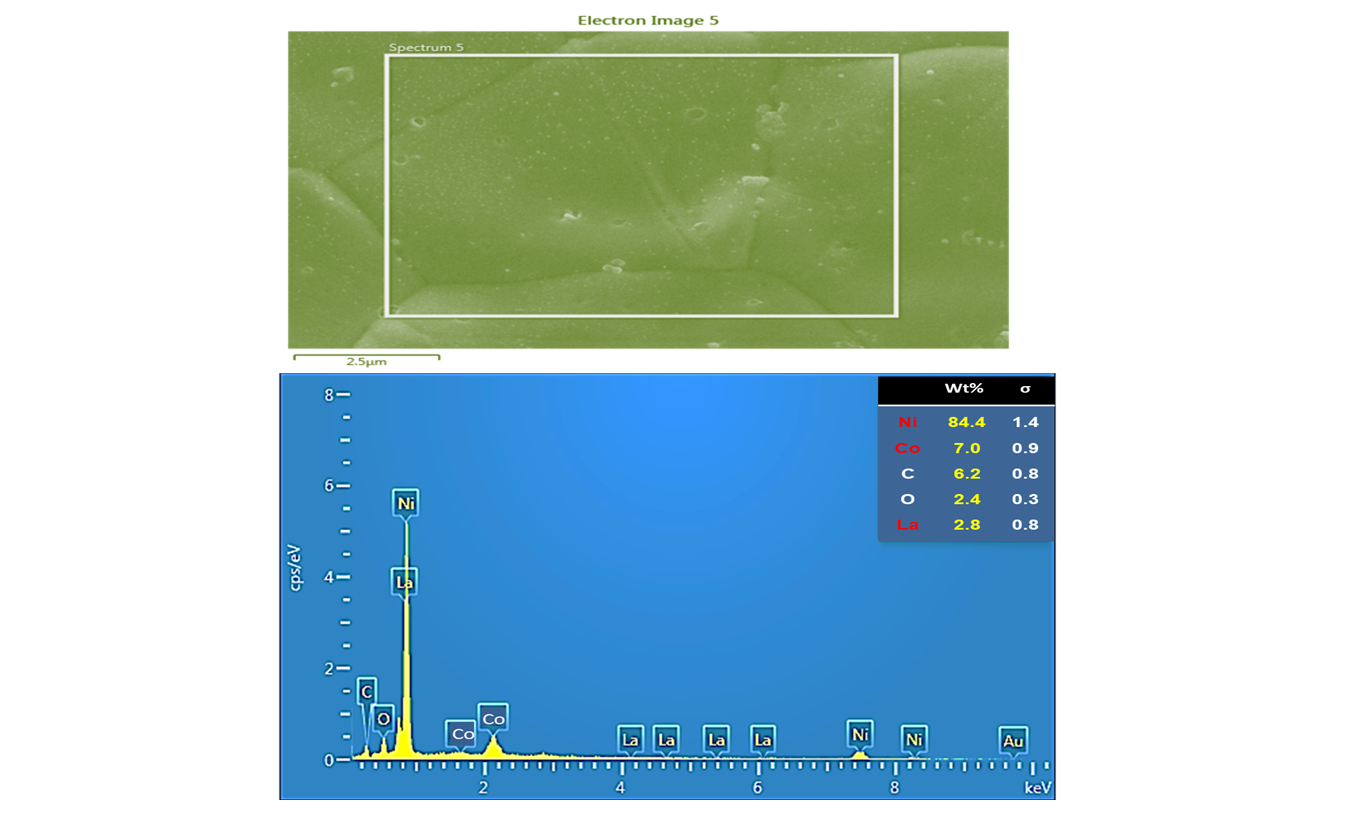


**Fig S1:** EDAX analysis of La-Co LDH.

**Supplementary Fig 2:**





**Fig 2:** (a) CV cyclic voltammetry of La-Co LDH in alkaline electrolyte(2M KOH). (b) Galvanostatic charge discharge measurement at various current density in alkaline electrolyte (2M KOH). (c) EIS measurement of La-Co LDH in alkaline electrolyte (2M KOH). (d) % rate capability of electrode materials in alkaline electrolyte (2M KOH).

**Supplementary Fig S3:**


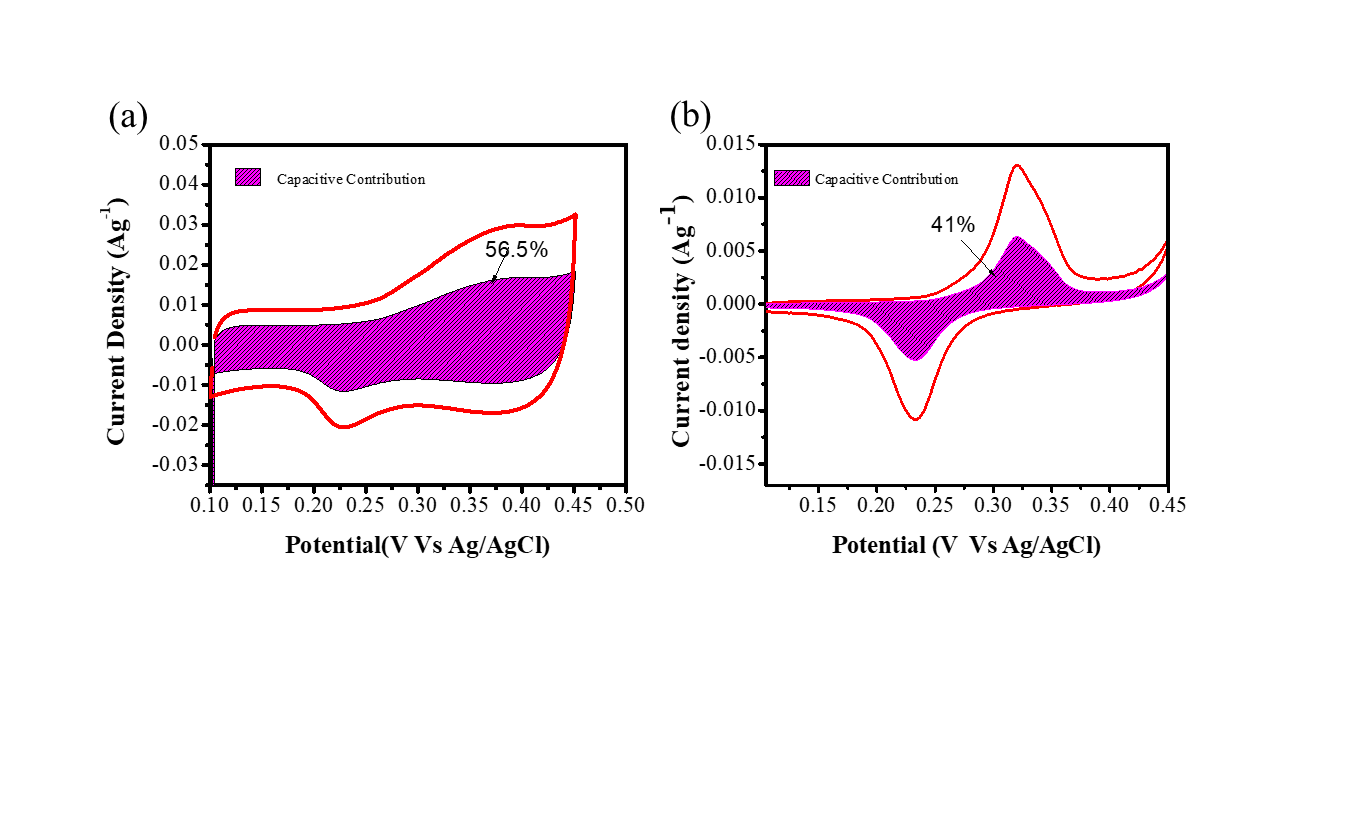


**Fig S3:** CV profile of (a) La-Co LDH and (b) Co(OH)_2_ at the scan rate of 10mVs^-1^, the estimated capacitive response of electrode materials is shown in shaded region.

**Supplementary Fig S4:**

**
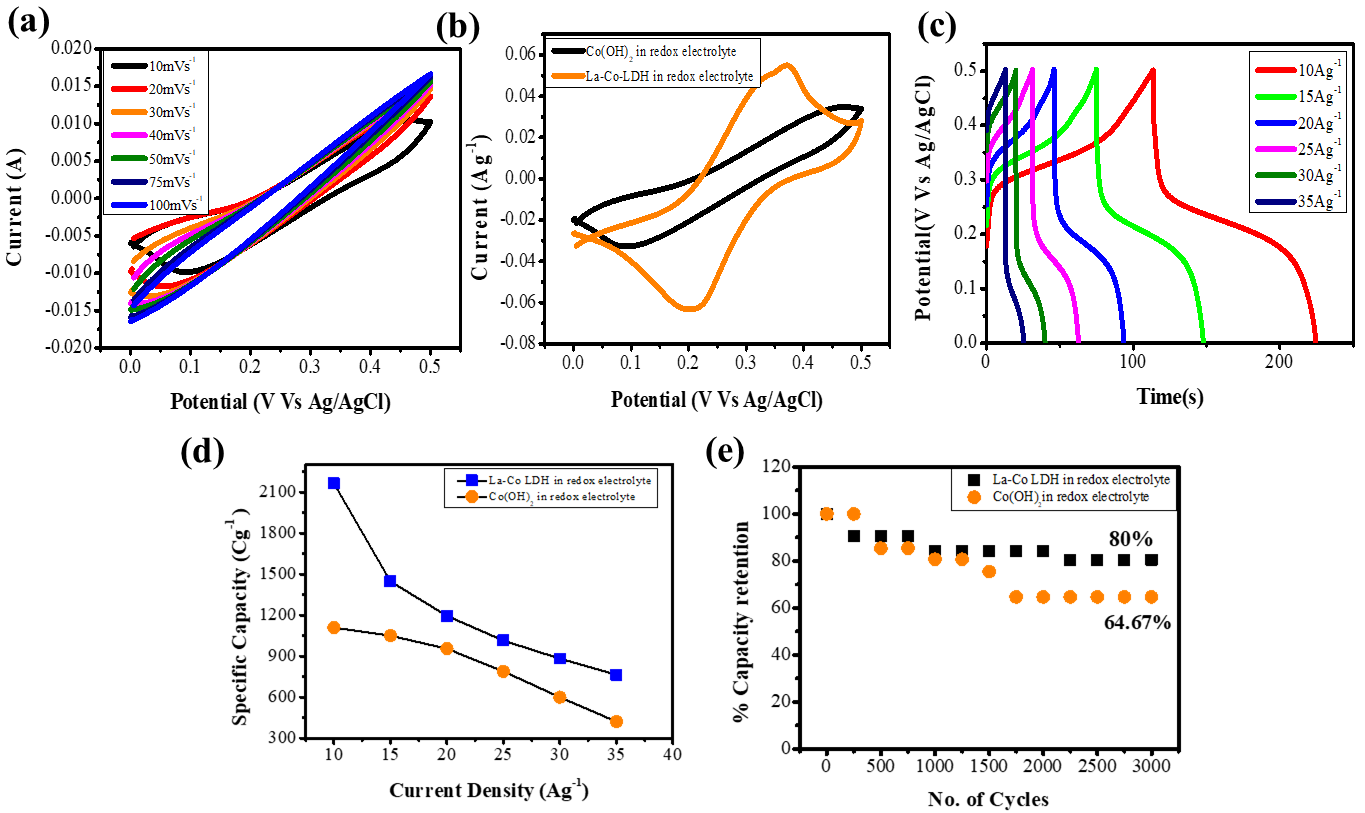
**

**Fig S4**. (a) Cyclic voltammetry analysis of Co(OH)_2_ in redox electrolyte, (b) comparative cyclic voltammetry analysis of La-Co LDH with Co(OH)_2_ in redox electrolyte,(c) Galvanostatic charge discharge plot of Co(OH)_2_ in redox electrolyte,(d) comparative specific capacity Vs current density plot of La-Co LDH with Co(OH)_2_ in redox electrolyte, ( e) comparative cyclic stability plot of La-Co-LDH with Co(OH)_2_ in redox electrolyte.

**Supplementary Fig S5:**





**Fig S5:** comparative specific capacity Vs current density plot of La-Co LDH in redox and alkaline electrolyte.

**Supplementary Fig S6:**

**
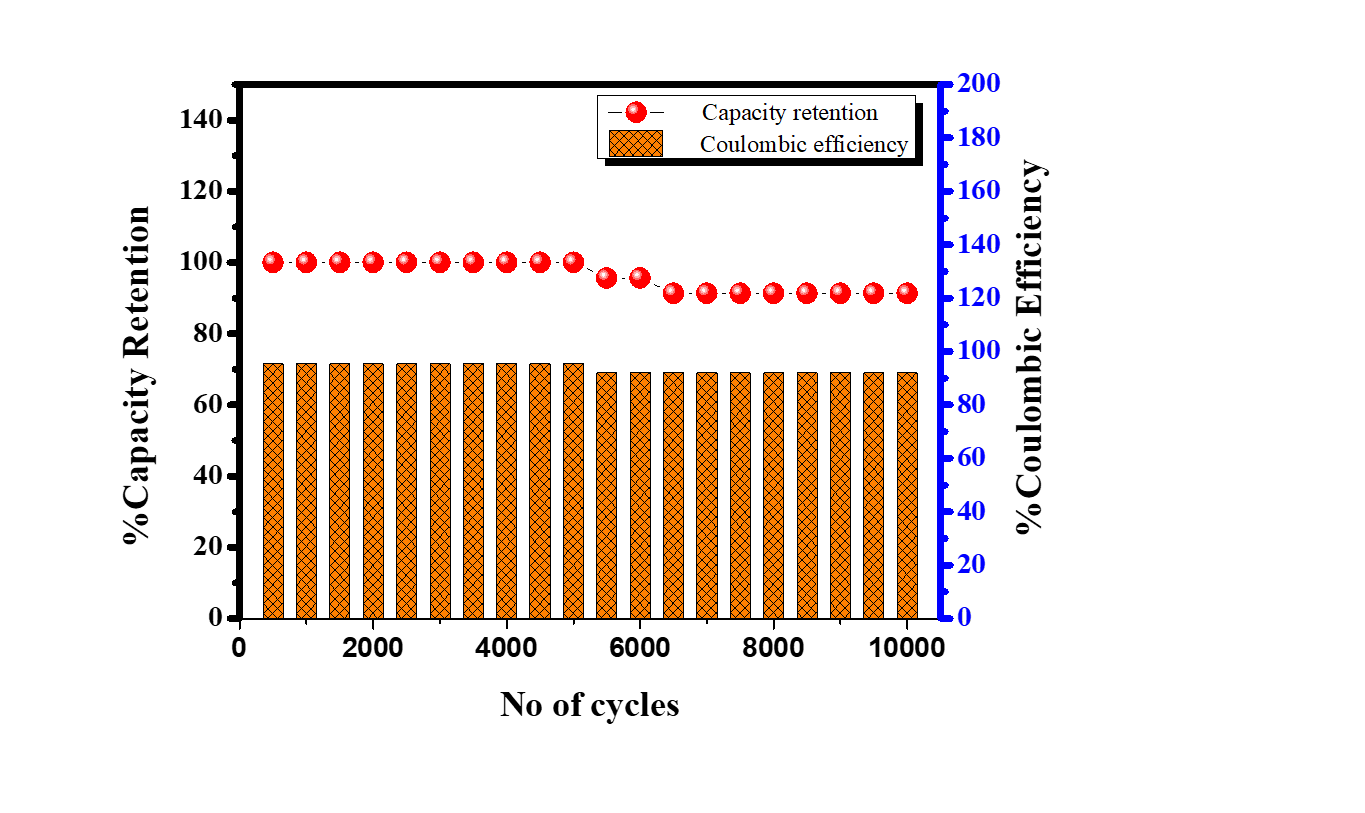
**

**Fig S6:** % cyclic stability plot of La-Co LDH electrode in alkaline electrolyte.

**Supplementary Fig S7:**


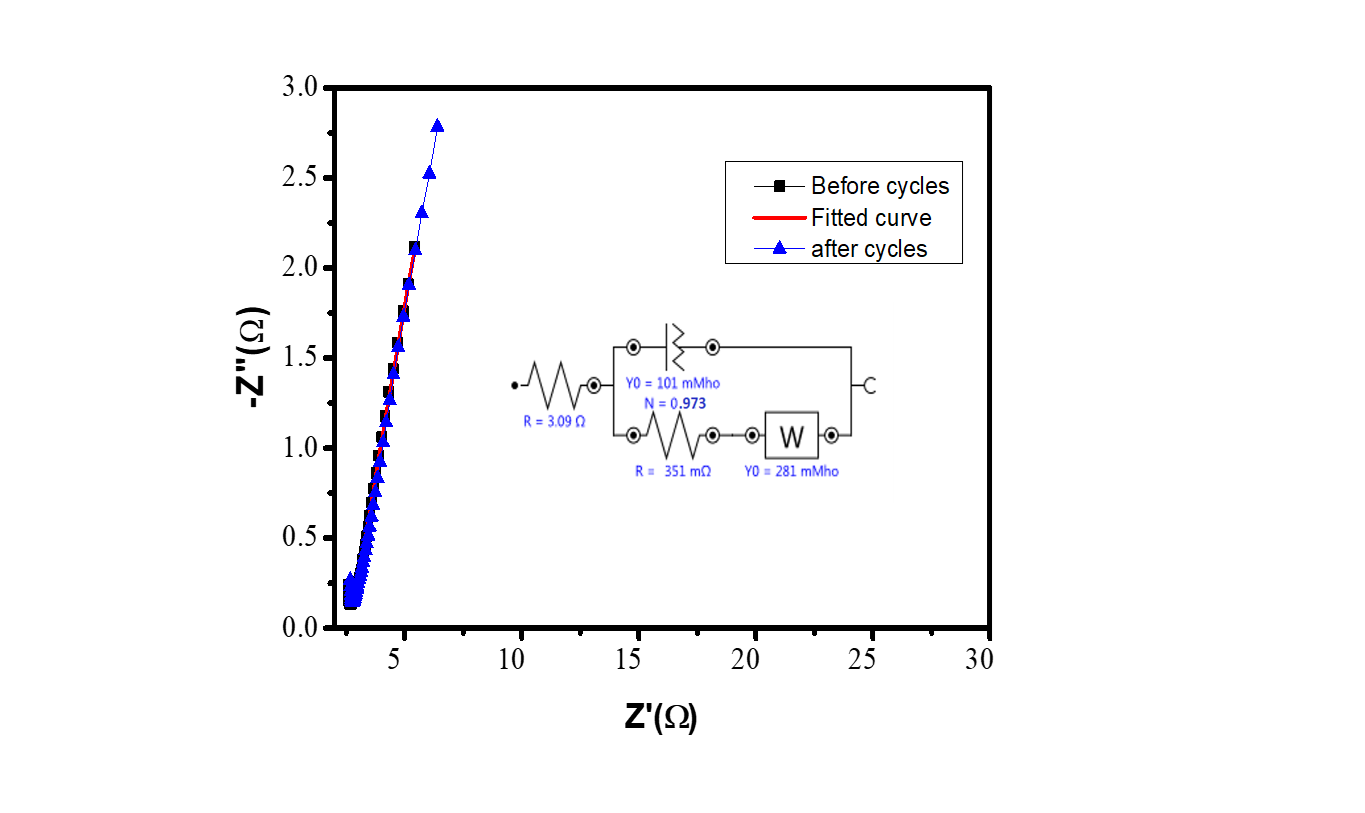


**Fig S7:** Nyquist plot of La-Co LDH nanosheet in redox electrolyte before and after cycles.

**Supplementary Fig S8:**


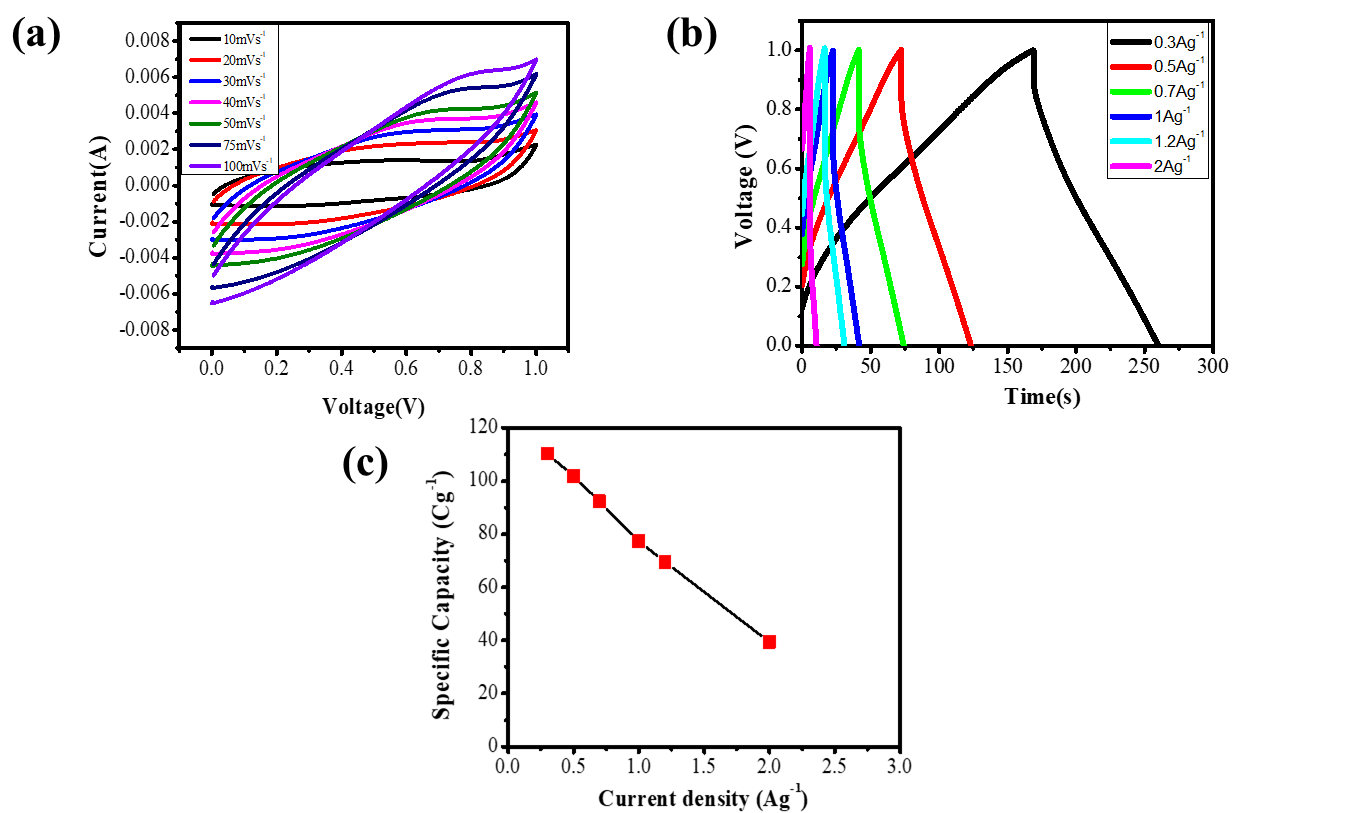


**Fig S8:**(a) Cyclic voltammetry curve of activated carbon cloth (ACC) electrode as symmetric electrochemical double layer supercapacitor,(b) Galvanostatic charge discharge (GCD) analysis of activated carbon cloth of symmetric electrochemical double layer supercapacitor, (c) Current density vs specific capacity of symmetrical ACC.

**Supplementary Fig S9:**


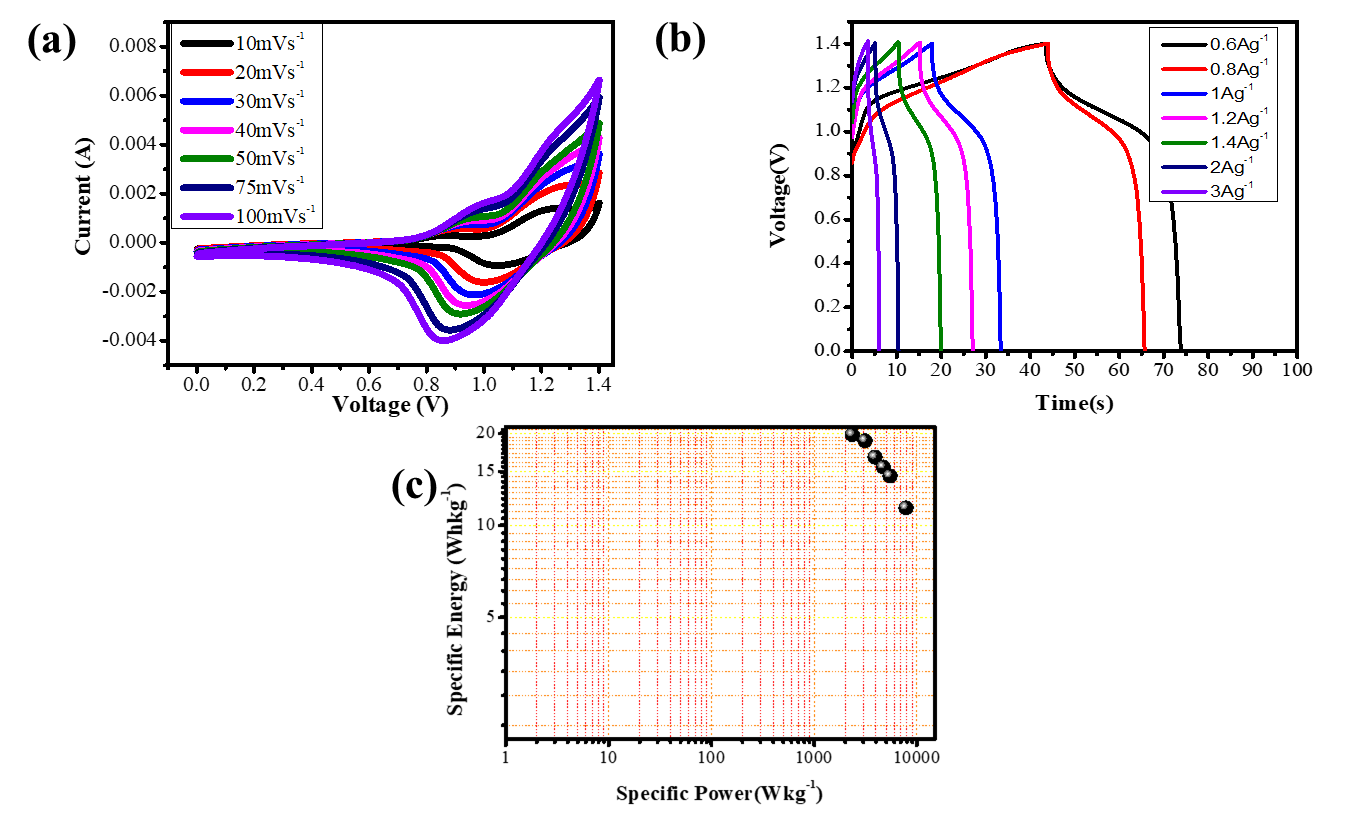


**Fig S9:**(a) Cyclic voltammetry curve of La-Co-LDH/CC in alkaline electrolyte,(b) Galvanostatic charge discharge analysis of La-Co LDH/CC in alkaline electrolyte,(c) Ragone plot of specific energy and power of La-Co LDH/CC in alkaline electrolyte.

**Supplementary Fig S10:**


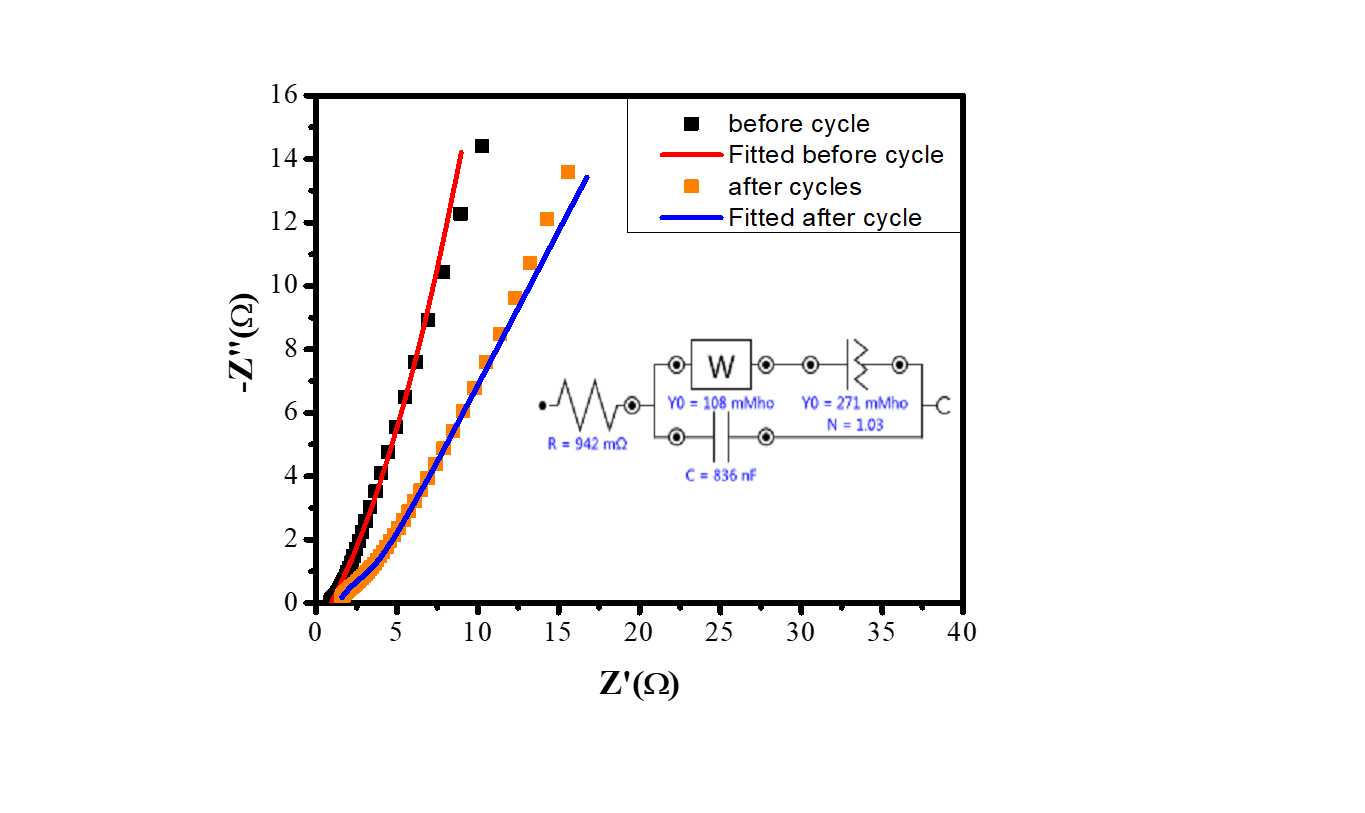


**Fig S10:** Nyquist plot of La-Co LDH/CC two electrode cell in redox electrolyte.

**Supplementary Table 1**

| Sr No | Current Density  Ag^-1^ | Specific Capacity  Cg^-1^ (Co(OH)_2_ in alkaline electrolyte | Specific Capacity  Cg^-1^ (La-Co LDH in alkaline electrolyte | Specific capacity Cg^-1^((Co(OH)_2_ in redox electrolyte | Specific capacity Cg^-1^( La-Co LDH in redox electrolyte) |
| --- | --- | --- | --- | --- | --- |
| 1 | 10 | 160 | 191.19 | 1108.4 | 2162.4 |
| 2 | 15 | 156 | 190.65 | 1050 | 1446.6 |
| 3 | 20 | 152 | 190.4 | 954.4 | 1194.36 |
| 4 | 25 | 150 | 184.99 | 788 | 1014 |
| 5 | 30 | 147 | 183.3 | 598.8 | 880.8 |
| 6 | 35 | 140 | 182 | 420.2 | 763 |

**Table T1:** Electrochemical performance of La-Co LDH, Co(OH)_2_ in alkaline and redox electrolyte

**Supplementary Table T2:**

| Electrode materials | Electrochemical performances | Cyclic stability | References |
| --- | --- | --- | --- |
| Ni-Co LDH/RGO | 875Cg^-1^ at 1Ag^-1^ | 2000 cycles with 80% retention | 1 |
| Ni-Co Al/LDH in V_4_C_3_ Mxene | 627 Cg^-1^ at1Ag^-1^ | 3000 cycles with 67.39% retention | 2 |
| Ni-Mn LDH | 846.5 Cg^-1^ at 1Ag^-1^ | 2000 cycles with 79% retention | 3 |
| Co Mn LDH/Ni Foam | 1409 Fg^-1^ at 1Ag^-1^ | 3000 cycles with 71.1 % retention | 4 |
| Ultrathin Ni_1-x_Co_x_S_2_ nanoflakes | 533.4Cg^-1^ at 0.5 Ag^-1^ | 3000 cycles with 57.11 % retention | 5 |
| La-Co LDH/Ni foam | 2162 Cg^-1^ at 10 Ag^-1^ | 3000 cycles with 80% retention | Present work |

**Table T2:** Performances of hydroxides pseudocapacitors.

**Supplementary Table T3:**

| Current density Ag^-1^ | Specific capacity Cg^-1^(La-Co LDH/CC in redox electrolyte) | Specific capacity Cg^-1^(La-Co LDH/CC in alkaline electrolyte) |
| --- | --- | --- |
| 0.6 | 218.04 | 72.67 |
| 0.8 | 200.32 | 69.50 |
| 1 | 184.80 | 61.44 |
| 1.2 | 174.70 | 57.02 |
| 1.4 | 166.63 | 53.31 |
| 2 | 142.72 | 41.92 |
| 3 | 111.39 | 30.24 |

**Table T3:** Performance comparison of La-Co LDH/CC in redox electrolyte and alkaline electrolyte.

**Supplementary Table** **T4:**

| Sr no | Current Density  Ag^-1^ | Specific Capacity  Cg^-1^ | Volumetric capacity  Ccm^-3^ | Energy Density  Whkg^-1^ | Power Density  Wkg^-1^ | Volumetric Energy density  mWhcm^-3^ | Volumetric power  mWcm^-3^ |
| --- | --- | --- | --- | --- | --- | --- | --- |
| 1 | 0.6 | 218.04 | 271.02 | 42.46 | 1687.15 | 52.69 | 2093.64 |
| 2 | 0.8 | 200.32 | 250.04 | 38.95 | 2239.93 | 48.61 | 2795.46 |
| 3 | 1 | 184.80 | 231.05 | 35.93 | 2799.74 | 44.92 | 3500.25 |
| 4 | 1.2 | 174.70 | 218.38 | 33.96 | 3358.95 | 42.46 | 4199.68 |
| 5 | 1.4 | 166.63 | 208.29 | 32.40 | 3920.67 | 40.50 | 4899.68 |
| 6 | 2 | 142.72 | 178.04 | 27.75 | 5599.77 | 34.61 | 6984.08 |
| 7 | 3 | 111.39 | 139.24 | 21.65 | 8395.99 | 27.07 | 10497.89 |
| 8 | 5 | 80.79 | 100.97 | 15.70 | 13993.56 | 19.63 | 17496.41 |

**Table T4**: Performance of two electrode ASC device (La-Co LDH/CC) in redox electrolyte.

**Supplementary note**

Experimental formula:

1. **Specific capacity of Three electrode system**^1-5^:

Qsp=$\frac{I\Delta t}{m}$ Cg^-1^ (1)

1. **Specific Capacity for two electrode Ce**ll^6-8^

Qsp= 4$\frac{C}{m}$(2)

= 4$\frac{I\Delta t}{m}$ Cg^-1^

As m =$\frac{m1m2}{m1+m2}$

Where, Qsp specific capacity of the device, I is the applied current, ∆t is the discharging time and m is the mass of the electrode material in asymmetric device

1. **Energy and power density in case of two electrode cell**^2,3,7^

- Esp= $\frac{{C0V0}^{2}}{2}$ , where C= Q_sc_/V_0_ (3)
- Psp= $\frac{E}{\Delta t}$

Where, Esp is the specific energy density of the ASC device, C is specific capacitance measured in terms of specific capacity, V_0_ is the voltage window. ∆t is the discharge time.

1. **Columbic efficiency:**

ɳ = $\frac{td}{tC}$× 100 (4)

Where, td is the discharging time, tc is the charging time.

Reference:

1. Liu, L., Liu, A., Xu, Y., Yu, H., Yang, F., Wang, J., Deng, S. Agglomerated nickel–cobalt layered double hydroxide nanosheets on reduced graphene oxide clusters as efficient asymmetric supercapacitor electrodes. Journal of Materials Research, 1–9 (2020).
2. Wang, X., Li, H., Li, H., Lin, S., Bai, J., Dai, J., … Dou, S. Heterostructures of Ni−Co−Al layered double hydroxide assembled on V_4_C_3_ MXene for high-energy hybrid supercapacitors. Journal of Materials Chemistry A**J. Mater. Chem. A** ,**7**, 2291-2300(2019).
3. Xiaoliang Wang, Jiaqi Zhang, Shaobin Yang, Huiyan Yan, Xiaodong Hong, Wei Dong, Yi Liu, Boyan Zhang, Zhen Wen, Interlayer Space Regulating of NiMn Layered Double Hydroxides for Supercapacitors by Controlling Hydrothermal Reaction Time, Electrochimica Acta, 18, 32223 (2018).
4. D. Chen, H. Chen, X. Chang, P. Liu, Z. Zhao, J. Zhou, G. Xu, H. Lin, S. Han, Hierarchical CoMn-layered double hydroxide nanowires on nickel foam as electrode material for high-capacitance supercapacitor, J. Alloys Compd. 729 866–873 (2017).
5. Xiaoxiang Wang, Teng Wang, Rusen Zhou, Lijuan Fan, Shengli Zhang, Feng Yu, Tuquabo Tesfamichael, Liwei Su and Hongxia Wang, Ultrathin Ni_1−x_CoxS_2_ nanoflakes as high energy density electrode materials for asymmetric supercapacitors, Beilstein J. Nanotechnol., 10, 2207–2216(2019).
6. Subramani, K., Sudhan, N., Divya, R., & Sathish, M. All-solid-state asymmetric supercapacitors based on cobalt hexacyanoferrate-derived CoS and activated carbon. RSC Advances, 7(11), 6648–6659 (2017).
7. Fei, H., Saha, N., Kazantseva, N., Moucka, R., Cheng, Q., & Saha, P. A Highly Flexible Supercapacitor Based on MnO_2_/RGO Nanosheets and Bacterial Cellulose-Filled Gel Electrolyte. Materials, 10(11), 1251 (2017).
8. Gao, X., Lv, H., Li, Z., Xu, Q., Liu, H., Wang, Y., & Xia, Y. Low-cost and high-performance of a vertically grown 3D Ni–Fe layered double hydroxide/graphene aerogel supercapacitor electrode material. RSC Advances, 6(109), 107278–107285 (2016).
